# Editorial: GBM stem cells and the brain tumor microenvironment

**DOI:** 10.3389/fonc.2023.1153803

**Published:** 2023-02-24

**Authors:** Yunqing Li, Roger Abounader

**Affiliations:** ^1^ Hugo W. Moser Research Institute at Kennedy Krieger, Baltimore, MD, United States; ^2^ Department of Neurology, The Johns Hopkins University School of Medicine, Baltimore, MD, United States; ^3^ Department of Microbiology, Immunology and Cancer Biology, University of Virginia, Charlottesville, VA, United States; ^4^ Department of Neurology, University of Virginia, Charlottesville, VA, United States; ^5^ University of Virginia National Cancer Institute (NCI)-Designated Comprehensive Cancer Center, Charlottesville, VA, United States

**Keywords:** GSC intrinsic mechanisms, GBM TME, GBM stem-like cells, GBM heterogeneity, interactions between GSCs and the GBM TME

A small subset of tumor cells has been identified in glioblastoma (GBM). These cells have the capacity for unlimited self-renewal, multi-lineage differentiation, chemotherapy/radiation resistance, and efficiently propagating tumors in xenograft models. An increasing number of genomic, epigenomic, transcriptomic profiling, and experimental studies have demonstrated that these stem-like tumor-propagating GBM cells (GBM stem cells, GSCs) are associated with the GBM heterogeneity and GBM immunosuppressive microenvironment (TME) leading to maintaining GBM growth, therapeutic resistance, and GBM recurrence. Understanding the interactions of GSCs and GBM heterogeneity and the GBM TME and targeting these interactions could have a tremendous impact on GBM treatment. However, our current understanding of how GSCs modulate GBM heterogeneity and GBM TME and their interactions is limited and inadequate. This Research Topic of Frontiers in Oncology focuses on understanding and targeting the interactions between GSCs and GBM cellular heterogeneity and GBM microenvironment. We collected six interesting articles including 2 original research papers and 4 review papers.

Two review articles and two original research articles focused on understanding GSC intrinsic genetic mechanisms contributing to the induction of GBM heterogeneity and GBM immunosuppressive microenvironment. They show and discuss GSCs as key contributors in recruiting pro-tumor immune cells, leading to the formation a GBM immunosuppressive microenvironment. The paper from Johnson et. al. comprehensively summarized recent advances in our understanding of how the drivers of GSC plasticity and heterogeneity (e.g., transcription factors, histone modifications, DNA and RNA methylation, metabolic reprogramming) modulate the GBM tumor immunosuppressive microenvironment (TIME). The paper also discussed recent advances in our understanding of GSC-intrinsic mechanisms that modulate GSC-TIME interactions and presented cutting-edge techniques and bioinformatics platforms that are available to study immune modulation at high cellular resolution for the exploration of both malignant (i.e., GSC) and non-malignant (i.e., immune) cell fractions. The article highlighted GSC-intrinsic mechanisms, including functional mimicry of immune suppressive cell types as determinants of anti-tumor immune escape and therapeutic approaches for targeting immunomodulatory GSC-intrinsic mechanisms to potentiate immunotherapy responses in gliomas. A similarly interesting paper from Silver et al. discussed the diversity of GSCs with distinct characteristics presented in GBM and how GSC diversity drives global intratumoral heterogeneity constituted by complex and spatially distinct local microenvironments. It also discussed how to map this intricate GBM ecosystem through the lens of metabolism and immunology to find vulnerabilities and new ways to disrupt the equilibrium of the system to achieve improved disease outcomes.

Two original research articles from Ren et al. and Yang et al., separately show that dysregulated gene expression in GSCs regulates immune cell infiltration and alters the GBM TME. Ren et al.’s paper showed that non-SMC condensin II complex subunit G2 (NCAPG2) was highly expressed in GSCs and knockdown of NCAPG2 significantly inhibited the self-renewal ability of GSC. The functional annotation showed that NCAPG2 was mainly involved in the immune response and the Wnt signaling pathway. Increased expression of NCAPG2 was correlated with infiltration levels of various immune cells and immune checkpoint in glioma. Yang et al.’s paper studied the biological function and clinical characterization of nine minichromosome maintenance protein members (MCM) in low-grade glioma using diverse public databases. The data showed that MCM family members were consistently up-regulated in glioma tissues and glioma cell lines and higher expression of MCM2-MCM8 and MCM10 was linked with poor overall survival (OS) and shorter disease-specific survival (DSS) in glioma patients. Functional enrichment analysis indicated that MCMs mainly participated in regulating cell cycle and DNA replication. DNA copy number variation (CNV) and DNA methylation significantly affect the expression of MCMs. Importantly, MCMs expression was highly correlated with immune cell infiltration, immune modulator, tumor mutational burden (TMB), and drug sensitivity.

Two other interesting review articles focus on understanding GSC non-genetic factors contributing to the regulation of GBM heterogeneity and GBM TME. The review paper by Wang et al. discussed a subpopulation of GBM cells with tumor microtubes (TMs) identified in GBM. This TM-positive GBM subpopulation expresses neural stem cell markers and shares many features with both immature neurons and cancer stem cells. The authors discussed the common features between TM and sprouting axons in morphology, formation, and function. This review article provides a new angle to understand the roles of TM-positive GBM cell subsets in resistance and recurrence mechanisms, the incurability and heterogeneity of gliomas, and current potential therapeutic strategies for targeting TM. Another review article by Akindona et al. discussed the relationship between the nonmalignant neural and hematopoietic stem cell niches and cancer stem cell niche, and hypothesized that the signaling pathways involved in the development and maintenance of the NSC and HSC niches may provide the development of the GSC niche. They discussed the role of angiogenesis as a potential component of GSC niche development and provide a hypothesis for its development in GBM. Targeting vessel co-option mechanisms may prove to be an effective strategy for targeting tumor vascularization, perhaps in conjunction with anti-VEGF therapy. The authors point out that a better understanding of the mechanisms of development of the tumor stem-like cell niche may provide new insights that could be amenable to therapeutic exploitation.

Altogether, this Research Topic of Frontiers in Oncology emphasizes GSC intrinsic mechanisms including genetic alterations (e.g. gene mutation, gene copy number alteration, gene translocation, and epigenetic modification, etc.) and non-genetic factors (e.g., tumor microtubes) that contribute to the induction of GBM heterogeneity and regulation of the GBM microenvironment including cellular and non-cellular components. The issue also describes advanced technologic platforms that are available to study dynamic interactions between GSCs and the GBM TME. The overall findings suggest new avenues for the therapeutic exploitation and targeting of the above interactions with new potential GBM therapies.

## Author contributions

Writing, Review, and Editing: YL and RA. All authors contributed to the article and approved the submitted version.

